# Risk Factors of Standalone and Coexisting Forms of Undernutrition Among Children in Sub-Saharan Africa: A Study Using Data from 26 Country-Based Demographic and Health Surveys

**DOI:** 10.3390/nu17020252

**Published:** 2025-01-11

**Authors:** Misganaw Gebrie Worku, Itismita Mohanty, Zelalem Mengesha, Theo Niyonsenga

**Affiliations:** 1 Health Research Institute, Faculty of Health, University of Canberra, Canberra, ACT 2617, Australia; itismita.mohanty@canberra.edu.au (I.M.); zelalem.mengesha@canberra.edu.au (Z.M.); theo.niyonsenga@canberra.edu.au (T.N.); 2 Department of Human Anatomy, School of Medicine, College of Medicine and Health Sciences, University of Gondar, Gondar P.O. Box 196, Ethiopia

**Keywords:** undernutrition, child anthropometric failure, coexisting undernutrition, sub-Saharan Africa

## Abstract

Introduction: Undernutrition in low- and middle-income countries (LMICs) remains a leading public health challenge. It accounts for one-third of the under-five mortality rate in sub-Saharan Africa (SSA). This study applied the composite index of anthropometric failure (CIAF) to assess the prevalence of various standalone and coexisting forms of undernutrition and identify associated risk factors. Methods: Nationally representative demographic health survey (DHS) data from 26 SSA countries were used. A multilevel multinomial logistic regression analysis was conducted considering the hierarchical nature of DHS data and more than two categories of outcome variable. Four models were fitted and the model with the highest log-likelihood and lowest deviance was chosen as the best-fitted model. The adjusted relative risk ratio (aRRR) with its corresponding 95% confidence interval (CI) was presented as a measure of the effect. Results: The overall prevalence of undernutrition among under-five children in SSA was 34.59% (95% CI: 34.35–34.82). Additionally, 20.49% (95% CI: 20.30–20.69) and 14.09% (95% CI: 13.92–14.26) of under-five children had standalone and coexisting undernutrition, respectively. The mother’s educational level and household wealth status were the most significant shared drivers for standalone and coexisting undernutrition. On the other hand, child and health service factors were differentiating factors between standalone and coexisting undernutrition. Age of the child, sex of the child, type of birth, birth weight, adherence to age-appropriate feeding, antenatal care visit (ANC), place of delivery, and maternal educational status were the most significant determinants of various undernutrition forms in 0–23-month-old children. For 24–59-month-old children, age of the child, sex of the child, type of birth, household wealth status, and maternal education were identified as the main determinants of different forms of undernutrition. Conclusions: Our analysis revealed that distal factors were shared risk factors among standalone and coexisting forms of undernutrition. However, proximal and intermediate factors varied in the type and strength of the association between standalone and coexisting undernutrition. This implies that holistic and category-specific strategies are needed to significantly reduce undernutrition among under-five children in SSA.

## 1. Introduction

A recent global nutrition report revealed alarming figures regarding undernourishment in children, manifested as stunting (148.1 million) and wasting (45 million). Despite some progress in reducing stunting (26% to 22.3%) and wasting (8% to 6.8%) from 2012 to 2022, most sub-Saharan African (SSA) countries are off-track when it comes to meeting 2030 sustainable nutritional targets [1]. This implies that combating the impacts of undernutrition in the region continues to be challenging.

While prevalent across all age groups, undernutrition has a significant impact on children in low- and middle-income countries (LMICs). Undernutrition contributes to 44.08% of acute respiratory tract infections (ARIs) and 27.96% of cases of diarrhea [2]. Additionally, prolonged undernutrition among children results in several long-term consequences, such as deficient cognitive development and low contribution to economic productivity [3]. In Africa, especially in SSA, children continue to suffer from undernutrition and its associated complications, comprising 28% of under-five mortality [3].

Child nutritional status is routinely assessed using World Health Organization (WHO) standard anthropometric indices: height for age, weight for age, and weight for height. These anthropometric measures, called conventional indices, have been used since the 1970s to define a child’s nutritional status [4]. However, undernourished children are often not deficient in just one of these anthropometric indices; they instead suffer from multiple deficiencies captured through two or more of these indices [5]. In other words, while these indices are used to classify undernourished children into different distinct forms, in reality, the deficiencies coexist. Therefore, when these indices are considered independent as separate measures of undernutrition, they fail to accurately depict the true magnitude and complexity of undernutrition among children in LMICs [5,6,7]. In addition, conventional methods fail to specifically identify which children are affected by standalone and coexisting undernutrition. Consequently, the epidemiology of coexisting undernutrition is yet to be investigated.

An anthropometric method called the composite index of anthropometric failure (CIAF) has been recently introduced to account for various subgroups of undernutrition, specifically identifying children affected by standalone and coexisting undernutrition [5]. The CIAF approach overcomes the limitation of the conventional undernutrition measurement by providing information on the overall magnitude of undernutrition (the aggregated CIAF) and mutually exclusive standalone, double, and triple sub-measures of undernutrition (the disaggregated CIAF). Many researchers and relevant organizations, including United Nations International Children’s Emergency Fund (UNICEF), have recently adopted the CIAF to report the overall magnitude and prevalence rates of various disaggregated patterns of child undernutrition [5]. This shows an increased interest in replacing traditional conventional metrics by the CIAF for assessing and reporting the burdens of child undernutrition at global and country levels [7].

Furthermore, research investigating risk factors of child undernutrition has been mostly based on conventional indices [4,8,9] which mask determinants for coexisting manifestations of undernutrition. Studies utilizing the CIAF focused primarily on aggregated forms of the CIAF; that is, they classified children as nourished versus not nourished (a binary classifier). However, treating the CIAF in a binary manner leads to information loss as it obscures the distinction between standalone and coexisting sub-measures of undernutrition [6]. This limits available evidence about risk factors associated with various standalone and coexisting forms of undernutrition.

Few previous studies have investigated risk factors for various standalone and coexisting categories of child undernutrition and identified both shared and category-specific risk factors [10,11].

The literature also indicates that risk factors of undernutrition can vary between younger (0–23 months) and older children (24–59 months) [12]. However, most previous studies have analyzed children aged 0–59 months as a single group, focusing only on risk factors common to this age range or concentrated on specific age subsets such as 0–23, 24–59, or 6–59 months [4,13]. This results in a lack of evidence on how risk factors differ across age groups within the same population. Therefore, this study, using data from the DHS of SSA countries, investigated how various risk factors influence different forms of undernutrition among younger and older children.

## 2. Materials and Methods

### 2.1. Data Sources and Study Population

Nationally representative DHS data from 26 SSA countries were used for this study. Children whose anthropometric status was recorded according to the 2006 WHO growth standards were included. Children with flagged z-scores (height for age z-scores below −6 SD or above +6 SD, weight for age z-scores below −6 SD or above +5 SD, or weight for height z-scores below −5 SD or above +5 SD) were excluded as these children were considered biologically implausible [14].

DHS surveys are cross-sectional surveys with multistage cluster sampling conducted using standard methodology to generate comparable data on health and health-related indicators across countries [14]. Data in DHS are organized into men, women, children, birth, and household records. This study used data recorded for under-five children known as kid’s record (KR) dataset. The DHS data are publicly available at the measure DHS program website (https://www.dhsprogram.com/Methodology/Survey-Types/DHS.cfm, accessed on 25 November 2023) upon reasonable request. Further information regarding the DHS is available elsewhere [14].

### 2.2. Variables of the Study

#### 2.2.1. Outcome Variable

Realizing the challenges in using conventional forms of undernutrition such as stunting, wasting, or underweight to assess the burden of undernutrition in a population, the outcome variable in this study was more comprehensive: the CIAF in its disaggregated form. Disaggregated CIAF includes standalone (stunting only, wasting only, and underweight only), double (stunting–underweight and wasting–underweight), and triple (stunting–wasting–underweight) forms, as well as the nourished categories, leading to 7 categories of outcome.

#### 2.2.2. Explanatory Variables

The updated UNICEF conceptual framework [15] on child undernutrition was used to categorize potential risk factors of undernutrition as proximal, intermediate, and distal factors. The UNICEF framework was developed as part of its strategy for improving global child nutrition. Following the UNICEF framework, covariates identified and considered in this study include the following: proximal factors: age of the child, sex of the child, type of birth, birth interval, birth order, birth weight, and adherence to age-appropriate feeding; intermediate factors: media exposure, maternal age, type of toilet facility, sources of drinking water, distance to water sources, household size, parity, number of under-five children, antenatal care visit (ANC), post-natal checkup (PNC), place of delivery, and distance to health facility; and distal factors: maternal educational status, household wealth status, maternal working status, community illiteracy level, community poverty level, and place of residence. Community-level variables such as community illiteracy and poverty were generated by aggregating individual-level factors (educational status and wealth status) at the community cluster level and categorized as low and high based on the median value.

### 2.3. Data Management

Data coding, editing, and cleaning were conducted for each country’s dataset to ensure standardization and prevent variable mismatches before appending. Stata version.18.0 software was used for data cleaning and coding. Covariates with missing data were first identified and assessed for the percentage and patterns of missingness. The DHS expert group identified the missing data in the DHS dataset as missing at random (MAR). Most variables with missing data had less than 5% missingness rates, which are considered negligible. The missingness among these variables was also checked against other variables and did not relate to any of them, leading to the assumption of missingness at random. Thus, complete cases were considered for these variables. However, multiple imputation was performed for the birth weight variable, as the percentage of missingness was 41%. In the DHS dataset, the missing data for birth weight was recorded as not weighed at birth. We fitted a logistic regression model for the birth weight variable by categorizing it as “0” for not missing and “1” for missing data. The regression output indicated that the missing value in birth weight was significantly associated with home delivery and rural residence. Thus, the likelihood of missing birth weight was explained by other observed variables (primarily by place of delivery and place of residence). The birth weight of most children who were delivered at home and residing in rural areas was not recorded.

To handle this missing data, first, we converted the dataset into a wide format using the “mi set wide” Stata command. Then we registered birth weight as an impute variable and other covariates as regular (imputation) variables followed by multiple imputation using “mi impute mlogit” command in Stata since the birth weight has three categories. Given the large sample size and following suggestions from the literature, at least five imputations are generally sufficient for reliable estimates and standard errors, so we conducted five imputations for the missing data.

### 2.4. Statistical Analysis

Considering the survey design (e.g., oversampling), the nested structure of DHS data and the presence of more than two response categories for the outcome variable, a multilevel (to account for nested structure), weighted (to restore sample representativeness), multinomial (categorical outcome) logistic regression approach was implemented to obtain appropriate statistical estimates [16]. Considering the risk factors for undernutrition might vary between age groups, even among those under five years old, this study conducted separate analyses for 0–23-month-old and 24–59-month-old children.

This hierarchical multinomial logistic regression analysis was implemented via a generalized structural equation modeling method with the logit link function and multinomial family using “nourished” as the reference category. Four models were constructed for the multinomial outcome variable for each age group. The first (null) model did not include any explanatory variables at the individual or community levels. However, it incorporated random effects at the community and country level to assess the extent of variation in undernutrition attributable to differences between communities and countries. The second and third models were adjusted separately for individual- and community-level factors. The final model was adjusted for both individual- and community-level factors. Model comparison was made using log-likelihood and deviance. A model with the highest log-likelihood and lowest deviance (−log-likelihood ratio (-2LLR)) was chosen as the best-fitted model.

For the null models, the country-, community-, and household-level random effects were fitted separately, and high clustering effects were identified at the country and community levels. As a result, the subsequent models only included country and community as random effects. Country and community level variance, intraclass correlation coefficient (ICC), and percentage change in variance (PCV) were calculated for each model.

To avoid excessive parameters and unstable estimates, stepwise regression using mlogit (backward selection) was first applied to select variables to be included in all the models. Multicollinearity was checked among the included covariates, and low variance inflation factors (VIFs) were observed with a mean VIF of 1.37 in both groups. Finally, the adjusted relative risk ratio with its corresponding 95% confidence interval was presented as a measure of effect.

### 2.5. Ethics

All analyses presented in this paper were based on nationally representative demographic and health survey data, a publicly available dataset that contains no identifiable information about survey participants. All ethical standards, including informed consent, were strictly observed during the survey. This study was approved by the University of Canberra Human Research Ethics Committee under approval number 13640.

## 3. Results

### 3.1. Sociodemographic Characteristics

A total weighted sample of 157,651 under-five children was included, with 67,797 aged 0–23 months and 89,854 aged 24–59 months. Nearly half of the children in both age groups were female. Additionally, nearly 65% of children in both age groups were rural residents. Over one-third of the children in both age groups had mothers who did not have an education. Most (77.46%) of the younger children did not receive age-appropriate feeding. Moreover, 50.41% and 47.49% of the younger children were from communities with high maternal illiteracy rates and high poverty levels, respectively (Table 1).

### 3.2. Magnitudes of Undernutrition Among Children in Sub-Saharan Africa

The overall prevalence of undernutrition among children aged 0–59 months was 34.59% (95% CI: 34.35–34.82). In addition, 20.49% (95% CI: 20.30–20.69) and 14.09% (95% CI: 13.92–14.26) of 0–59-month-old children had standalone and coexisting undernutrition, respectively.

Nearly one-third (31.56% (95% CI: 31.21–31.9)) of 0–23-month-old children experienced at least one form of undernutrition. In children aged 0–23 months, 18.31% (95% CI: 18.02–18.6) were affected by standalone forms, while 13.21% (95% CI: 12.99–13.51) were experiencing coexisting forms of undernutrition.

**Figure 1:**
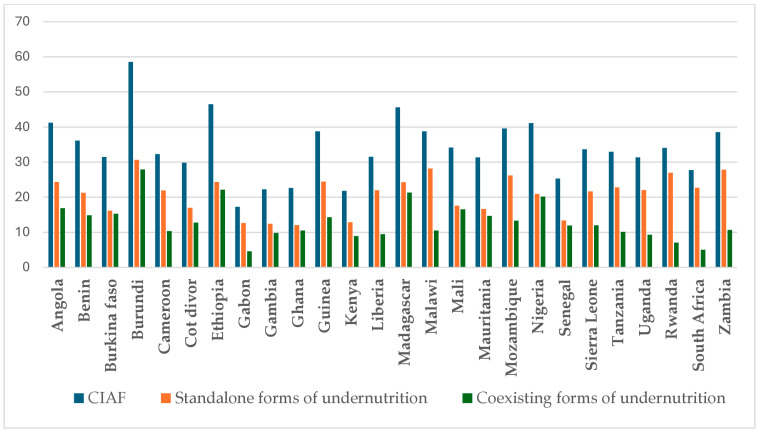
Prevalences of various forms of undernutrition by country in sub-Saharan Africa. Note: CIAF refers to the aggregate prevalence of undernutrition (composite of standalone and coexisting undernutrition). The standalone form of undernutrition is a composite of stunting only, wasting only, and underweight only. A coexisting form of undernutrition is a composite of stunting–underweight, wasting–underweight, and stunting–wasting–underweight.

Furthermore, 36.88% (95% CI: 36.56–37.20) of the 24–59-month-old children were affected by at least one form of undernutrition. Of these, 22.15% (95% CI: 21.88–22.42) and 15% (95% CI: 14.5–14.96) of children had standalone and coexisting undernutrition, respectively. In younger and older children, stunting only and the coexistence of stunting–underweight were the two most prevalent forms in the CIAF (Table 2 and Figure 1).

### 3.3. Random Effects and Model Fitness

Table 3 below presents the results of the random effect component of each model. For younger children, the ICC values from the null model revealed that 15.76% of the total variation in undernutrition was attributed to the clustering effect at the country level (3.26%) and community level (12.5%). The remaining 84.24% of the total variability in undernutrition among younger children was attributed to individual differences. Similarly, among older children, the ICC values indicate that 22.2% of the variation in undernutrition was attributed to differences in country-level (7.1%) and community-level (15.1%) clustering. On the other hand, the remaining unexplained 77.8% of the total variability in child undernutrition was attributed to individual-level differences.

As presented in Table 3, the percentage change in variance in the final model indicates that the incorporated individual- and community-level factors explained large proportions of the variation in undernutrition. As a result, 45% of the variation in undernutrition among younger children and 51% of the undernutrition variation among older children, observed in the null model, was explained by the included individual- and community-level factors.

### 3.4. Determinants of Undernutrition in Children Aged 0–23 Months

#### 3.4.1. Shared Risk Factors Across All Forms of Undernutrition in Younger Children

As shown in Table 4, maternal education and sex of the child were shared determinants among all standalone and coexisting forms of undernutrition in children aged 0–23 months. The female sex was associated with a lower risk of experiencing any form of undernutrition among younger children. The gender difference in child undernutrition was more decisive in coexisting forms of undernutrition, particularly in triple forms in which the female sex had a 61% (RRR = 0.39; 95% CI: 0.34–0.45) lower risk of experiencing stunting–wasting–underweight. Similarly, children with mothers who had no or minimal education, as well as those from communities with high illiteracy rates were more likely to experience both standalone and coexisting undernutrition.

#### 3.4.2. Determinants of Coexisting Undernutrition in Younger Children

A set of common child and health service factors predominantly influenced coexisting forms of undernutrition among younger children. The most significant risk factors include the age of the child, type of birth, birth weight, adherence to age-appropriate feeding, number of ANC visits, and place of delivery (Table 4).

Children aged 9–23 months had a higher risk of coexisting stunting–underweight (RRR = 2.27; 95% CI: 1.86–2.75), wasting–underweight (RRR = 1.61; 95% CI: 1.19–2.18), and triple forms of undernutrition (RRR = 5.75; 95% CI: 3.82–8.75) compared to children aged 0–5 months. Additionally, children born with a low birth weight were more likely to experience stunting–underweight (RRR = 2.22; 95% CI: 1.77–2.77), wasting–underweight (RRR = 1.95; 95% CI: 1.42–2.69), and triple forms of undernutrition (RRR = 2.2; 95% CI: 1.52–3.22) compared to those with a normal birth weight. Moreover, being a twin/multiple births was strongly associated with an increased risk of coexisting stunting–underweight (RRR = 5.21; 95% CI: 4.09–6.62) and stunting–wasting–underweight (RRR = 7.32; 95% CI: 5.53–9.68).

Conversely, adherence to age-appropriate feeding, having four or more ANC visits, and delivering in a health facility were associated with a reduced risk of coexisting undernutrition. Children who had age-appropriate feeding had a 21% lower risk of coexisting stunting–underweight (RRR = 0.79; 95% CI: 0.67–0.93) and a 32% lower risk of experiencing coexisting stunting–wasting–underweight (RRR = 0.68; 95% CI: 0.48–0.96).

#### 3.4.3. Risk Factors of Standalone Undernutrition in Younger Children

Child-related factors such as birth weight, age, and type of birth were major contributing factors to standalone undernutrition. The strength of associations of these factors with standalone undernutrition was relatively weak compared to the coexisting forms (Table 4).

Low-birth-weight children had a 49% greater risk of stunting only (RRR = 1.49; 95% CI: 1.26–1.77) and a 47% higher chance of experiencing underweight only (RRR = 1.47; 95% CI: 1.03–2.12) compared to normal-birth-weight children. In contrast to the coexisting forms, the age of the child showed a mixed association with standalone undernutrition. Specifically, children aged 9–23 months were 2.8 times more likely to experience stunting only (RRR = 2.8; 95% CI: 2.41–3.25). However, they had a 26% lower chance of experiencing wasting only (RRR = 0.74; 95% CI: 0.64–0.86), compared to those aged 0–5 months. Furthermore, being a twin/multiple births was associated with higher odds of stunting only (RRR = 2.56; 95% CI: 1.92–3.42) and underweight only (RRR = 2.92; 95% CI: 1.76–4.9). Overall, in children aged 0–23 months, child and health service factors were key differentiators between standalone and coexisting forms of undernutrition.

### 3.5. Determinants of Undernutrition in Children Aged 24–59 Months

#### 3.5.1. Common Risk Factors Across All Forms of Undernutrition in Older Children

Maternal educational level and household wealth status were shared determinants among standalone and coexisting forms of undernutrition in children aged 24–59 months. An improvement in wealth status from the poorest to richest consistently reduces the risk of experiencing any standalone and coexisting undernutrition. On the contrary, low levels of maternal education and living in communities with high maternal illiteracy rates were associated with higher odds of various forms of undernutrition, with a relatively stronger association observed among coexisting forms (Table 5).

#### 3.5.2. Determinants of Coexisting Undernutrition in Older Children

As presented in Table 5, proximal factors such as the sex of the child and type of birth were shared across all coexisting forms of undernutrition. The risk of experiencing each of the three coexisting forms of undernutrition (stunting–underweight (RRR = 0.89; 95% CI: 0.83–0.96), wasting–underweight (RRR = 0.85; 95% CI: 0.77–0.93), and stunting–wasting–underweight (RRR = 0.65; 95% CI: 0.57–0.74)) was significantly lower among female children compared to their male counterparts. Compared to children who did not have siblings born at the same time, higher risks of coexisting undernutrition forms were observed among children who were twins or whose mothers gave birth to multiple children at once (stunting–underweight (RRR = 2.45; 95% CI: 1.91–3.13), wasting–underweight (RRR = 1.65; 95% CI: 1.13–2.43), and stunting–wasting–underweight (RRR = 2.48; 95% CI: 1.71–3.62)).

#### 3.5.3. Risk Factors of Standalone Forms of Undernutrition in Older Children

The age of the child, sex of child, type of birth, and working status were the main determinants of standalone forms of undernutrition in older children.

Children of the female sex had a 19% lower risk of stunting only (RRR = 0.81; 95% CI: 0.77–0.85) but a 1.36 times higher risk of experiencing underweight only (RRR = 1.36; 95% CI: 1.18–1.58). Additionally, increases in age of the child were associated with reduced risks of both stunting only (RRR = 0.82; 95% CI: 0.77–0.87 for 36–47-month-olds and RRR = 0.55; 95% CI: 0.51–0.59 for 48–59-month-olds) and wasting only (RRR = 0.74; 95% CI: 0.59–0.91 for 36–47-month-olds) (Table 5). Further analysis results for model I (including only individual level factors) and model II (including only community level factors) are presented in the Supplementary File S1 (Tables S1–S4).

## 4. Discussion

This study applied the CIAF to estimate the prevalences of various standalone and coexisting forms of undernutrition and investigate whether proximal, intermediate, and distal factors affect standalone and coexisting undernutrition differently among under-five children in SSA. This study found that the overall prevalence of undernutrition among under-five children was 34.59%. Moreover, 20.49% and 14.09% of under-five children in SSA exhibited standalone and coexisting forms of undernutrition, respectively. The overall prevalence of undernutrition (34.59%), as well as the prevalences of coexisting undernutrition (14.09%) in this study, were lower than in findings from Bangladesh and India [10,11,17]. This difference may be attributed to our study’s use of multicounty data, in contrast to other studies that might overestimate the prevalence of undernutrition. However, the prevalence of standalone undernutrition in SSA (20.49%) was higher than reports from Bangladesh and India [10,11,17]. The high prevalence of undernutrition in SSA may be primarily and likely due to the widespread civil conflicts and the severe impacts of climate change and drought, which significantly hinder food production and access in the region [18]. Variations in dietary patterns, habits, and nutritional beliefs such as food taboos that prevent children from eating animal products, eggs, and chicken can also contribute to these undernutrition variations [19].

Our results revealed that maternal educational level and household wealth status were shared risk factors between standalone and coexisting undernutrition. Maternal education was the most significant driver of coexisting and standalone undernutrition in both 0–23- and 24–59-month-old children. Supported by other studies [11,20,21], this study found that children with mothers who had no or minimal education had an increased risk of experiencing standalone and coexisting undernutrition compared to those whose mothers had completed secondary school or higher levels of education. Similarly, children whose mothers were from a community with high illiteracy levels exhibited higher odds of both standalone and coexisting forms of undernutrition. This can most likely be due to higher levels of maternal education positively influencing the mothers’ awareness and ability to breastfeed, initiate complementary feeding, and engage in health-seeking behaviors [22].

Likewise, this study’s findings supported the previous literature [21,23,24] showing that an improvement in household wealth was associated with reduced risks of experiencing standalone and coexisting forms of undernutrition. This association of household wealth status with undernutrition was found to be substantially weak among 0–23-month-old children. Being from a low socioeconomic status (SES) limits the household’s financial capability to afford adequate and quality nutritional products [25]. Moreover, children with a low SES are more likely to be in poor household living conditions and exposed to different disease conditions which further increase the risks of undernutrition [10]. Therefore, these findings suggest that targeting broader socioeconomic factors, particularly initiatives to improve maternal education and household wealth, is instrumental in eradicating undernutrition in all its forms, one of the critical strategies of the sustainable development goals [26].

Among proximal factors, the sex of the child was found to be the main biological determinant of child undernutrition, in which the female sex was a protective factor in most cases. While female children in both age groups were less likely to experience coexisting undernutrition, gender differences in standalone forms of undernutrition were inconsistent. Specifically, for children aged 24–59 months, the female sex was associated with a lower risk of stunting only but a higher risk of underweight only. Some studies have reported the female sex as a protective factor against child undernutrition [27,28] while others have identified it as a risk factor [29]. In general, gender differences in child undernutrition are more consistent in severe or coexisting cases, with males being more likely to be adversely affected. Socio-cultural-related preferences for either sons or daughters result in gender inequities in childcare practice. In South Asia and North Africa, there is a strong preference for sons, while some societies in the Caribbean and SSA favor daughters. This could lead to a high undernutrition rate in the less-favored gender [10,29]. However, biological factors like body size and genetic differences in the immune system result in higher undernutrition rates among male children [28].

Our study highlighted that the age of the child was crucial in explaining coexisting and standalone forms of undernutrition, with the odds peaking at 9–23 months for most undernutrition forms. From six months of age onward, breastmilk alone does not provide all of a child’s nutritional requirements. During this period, children face unsafe complementary feeding practices [30] and increased rates of common childhood illnesses owing to dietary changes [31]. Coexisting and chronic undernutrition manifest after prolonged nutritional deficiencies, commonly at around the age of 12 months [21]. However, wasting only, a purely acute forms of undernutrition, tends to become evident at an earlier age. This demonstrates that the likelihood of experiencing different forms of undernutrition can significantly vary with age.

Twin/multiple births showed a stronger and more consistent association with coexisting undernutrition in both age groups compared to standalone undernutrition. This was consistent with studies conducted in Nigeria [13] and India [32]. The food competition among twins and children with multiple siblings born at once might cause insufficient nutrition to be obtained from breastmilk, which increases their likelihood of having various forms of undernutrition simultaneously [33].

Birth weight, adherence to age-appropriate feeding, place of delivery, and number of ANC visits, which are specific risk factors for children aged 0–23 months, disproportionately impacted standalone and coexisting forms of undernutrition.

Our results revealed that younger children born with a low birth weight experienced higher odds of stunting only, wasting only, and all coexisting forms of undernutrition compared to normal-birth-weight children. These findings were consistent with previous reports [11,34]. Interestingly, while estimating the magnitude of the effects of low birth weight on standalone and coexisting undernutrition, our study found a relatively strong association with coexisting forms of undernutrition. This is likely because children born with a low birth weight have small increments in their height and weight due to intrauterine growth restriction and altered growth-regulating mechanisms. This puts developing children at a heightened risk of experiencing multiple forms of undernutrition simultaneously [11].

Adherence to age-appropriate feeding was significantly associated with a reduced risk of stunting–underweight and stunting–wasting–underweight in children aged 0–23 months. This finding corroborates with a report from another study [35]. This is because adherence to age-appropriate feeding practices is established as nutritionally safe and adequate to meet the nutrient requirements necessary for younger children’s growth [36].

Regarding health service factors, this study found that younger children of mothers who had four or more ANC visits and who were delivered in health facilities had a lower chance of experiencing coexisting forms of undernutrition. The findings were consistent with other studies [20,25]. Mothers who attended the WHO-recommended four or more ANC visits and who delivered in a health facility are more likely to be well advised on child nutrition and healthcare services, leading to healthier food choices and better health-seeking behaviors [37]. This implies that improving ANC services and health facility delivery can effectively enhance a child’s nutritional status.

In general, this study, applied the CIAF approach, identified several statistically significant risk factors and demonstrated factors with a higher effect size. These factors can be considered the main contributing factors of undernutrition, influencing both standalone and coexisting forms and represent significant determinants across proximal, intermediate, and distal levels of the UNICEF’s framework. This information provides valuable insights into risk factors for different forms of undernutrition and could inform the design of more targeted and evidence-based nutrition policies, programs, and treatment guidelines. However, the CIAF approach requires further refinement or adaptation to enhance its relevance and practicality for nutrition programs and interventions. Additionally, since many health and nutrition programs currently depend on conventional undernutrition indicators, integrating the CIAF would necessitate adjustments to existing nutrition monitoring and evaluation systems, potentially requiring intensive resources and skilled personnel.

Moreover, distal factors were the shared risk factors influencing standalone and coexisting forms of undernutrition. However, most proximal and intermediate factors disproportionately influence standalone and coexisting undernutrition, with risk factors differing in type and strength of association. These differences in risk factors would also raise concerns about the effectiveness of existing nutrition services and interventions in addressing children with coexisting undernutrition, as they are typically based on conventional definitions of undernutrition. Our findings suggested that nutrition programs and initiatives targeting under-five children should consider coexisting forms of undernutrition and differences in risk factors between standalone and coexisting undernutrition to mitigate the burden of undernutrition in SSA.

### Strength and Limitation of the Study

This study has several strengths. First, it used a large amount of nationally representative DHS data from 26 countries in SSA and provided a more robust estimate of child undernutrition using a separate analysis for younger and older children. Moreover, this study was the first to comprehensively present risk factors for all standalone and coexisting undernutrition using disaggregated patterns of the CIAF in SSA. Hence, the findings can be generalized for countries in SSA. Multiple imputation was performed considering the percentage and pattern of the missing data to enable the maximal use of data for variables with large missing values.

The major limitation of this study arises from the cross-sectional nature of the DHS data, making it challenging to infer a cause-and-effect relationship between undernutrition and the included covariates. Another important limitation arises from recall bias, as the information for most of the DHS variables was collected through self-reporting. However, the DHS employs check and control mechanisms to ensure the accuracy of data collected across countries [38]. Consequently, recall bias is unlikely to impact the reliability of our estimates. Information regarding maternal nutritional status and dietary diversity were not collected for all countries of SSA, and those variables were excluded from these analyses. Moreover, the impacts of most environmental and system-level factors on child undernutrition were not assessed due to the absence of this information in the DHS data, which might have altered the results. We strongly recommend including these factors in future demographic and health surveys.

## 5. Conclusions

In conclusion, this study identified shared and category-specific determinants of various forms of child undernutrition. These findings will enable policymakers and program planners to implement a dual strategy, such as a holistic approach addressing shared drivers of undernutrition and targeted interventions addressing risk factors specific to various forms of undernutrition. Moreover, our findings strongly suggest the need to assess the extent of existing nutrition interventions and programs to effectively alleviate the burdens of coexisting undernutrition since existing nutrition interventions and programs are solely based on conventional undernutrition estimates. Finally, like the standalone forms of undernutrition, the assessment and reporting of coexisting undernutrition should be incorporated in standardized national nutrition surveillance and demographic health survey programs.

## Figures and Tables

**Table 1:** Sociodemographic characteristics of under-five children in sub-Saharan Africa.

Independent Variables		0–23-Month-Old Children		24–59-Month-Old Children	
		Frequency	Percentage (Weighted)	Frequency	Percentage (Weighted)
Age of the child in months	0–5	17,847	25.84		
	6–8	9140	13.26		
	9–23	42,054	60.90		
	24–35			30,977	33.57
	36–47			30,984	34.21
	48–59			29,645	32.22
Sex of child	Male	35,022	50.42	46,150	50.66
	Female	34,019	49.58	45,456	49.34
Birth order	First	15,547	22.77	19,340	21.54
	2nd–4th	33,804	49.27	45,066	49.68
	5th and above	19,690	27.96	27,200	28.78
Birth interval	No prior birth	15,547	23.00	19,538	21.74
	Less than 33 months	44,937	65.31	32,756	34.90
	33 months and above	8403	11.69	39,312	43.36
Type of birth	Single birth	66,768	96.79	88,744	96.93
	Multiple births	2273	3.21	2862	3.07
Birth weight	Low	7549	10.63		
	Normal	56,537	82.16		
	Macrosomia	4955	7.21		
Age-appropriate feeding	No	52,573	77.46		
	Yes	14,999	22.54		
Maternal age	15–24	24,222	34.42	19,539	21.04
	25–34	31,410	46.08	45,777	50.62
	35–49	13,409	19.50	26,290	28.35
Parity	Primiparous	15,062	22.06	9270	10.67
	Multiparous	34,028	49.62	48,931	53.93
	Grand multiparous	19,951	28.33	33,405	35.40
Maternal working status	Not working	31,184	44.40	36,367	38.61
	Working	37,857	55.60	55,239	61.39
ANC visit	No	6166	9.40		
	1–3	21,431	32.05		
	4 and above	38,338	58.55		
Postnatal checkup	No	49,446	74.04		
	Yes	17,353	25.96		
Place of delivery	Home	18,367	25.82		
	Health facility	50,674	74.18		
Media exposure	No	24,724	34.11	32,809	34.00
	Yes	44,317	65.89	58,797	66.00
Sources of drinking water	Not improved	35,562	50.39	46,712	50.06
	Improved	33,479	49.61	44,894	49.94
Distance to water source	On premise	20,711	32.71	27,705	32.59
	30 min and less	34,059	50.31	45,186	49.77
	Above 30 min	12,059	16.98	16,642	17.65
Distance to health facility	Big problem	25,300	38.48	33,817	38.65
	Not big problem	39,408	61.52	52,103	61.35
Type of toilet facility	Not improved	36,516	50.79	48,917	51.38
	Improved	32,525	49.21	42,689	48.62
Household size	4 and less	18,001	26.77	20,838	23.79
	5–9	37,775	55.00	53,738	58.66
	10 and above	13,265	18.23	17,030	17.55
Number of children under five	Below 3	49,812	73.48	67,022	74.60
	3 and above	19,229	26.52	24,584	25.40
Household wealth status	Poorest	18,054	22.65	24,336	22.80
	Poorer	14,933	21.35	19,435	21.11
	Middle	13,971	20.35	18,320	19.94
	Richer	11,932	18.91	16,094	19.27
	Richest	10,151	16.74	13,421	16.87
Maternal educational level	No education	25,978	36.25	37,922	39.87
	Primary	21,820	31.93	29,228	32.06
	Secondary and above	21,243	31.83	24,456	28.07
Place of residence	Rural	22,837	34.67	61,474	65.46
	Urban	46,204	65.33	30,132	34.54
Community illiteracy level	Low	33,235	49.59	45,776	51.62
	High	35,806	50.41	45,830	48.38
Community poverty level	Low	33,543	52.51	44,717	52.66
	High	35,498	47.49	46,889	47.34
Year of survey	Before COVID-19	36,048	53.29	47,624	52.95
	During COVID-19	32,993	46.71	43,982	47.05

Notes: ANC = Antenatal care.

**Table 2:** Prevalences of various standalone and coexisting forms of undernutrition in sub-Saharan Africa.

CIAF Categories	Weighted Prevalence of Undernutrition (95% CI)		
	0–23 Months	24–59 Months	0–59 Months
No failure	68.44 (68.09–68.78)	63.12 (62.79–63.43)	65.41 (65.17–65.64)
Stunting only	14.50 (14.23–14.76)	19.53 (19.27–19.79)	17.36 (17.18–17.55)
Wasting only	2.48 (2.36–2.59)	1.53 (1.45–1.60)	1.93 (1.86–2.01)
Underweight only	1.33 (1.24–1.42)	1.09 (1.02–1.16)	1.19 (1.14–1.25)
Stunting–Underweight	8.03 (7.82–8.23)	11.65 (11.44–11.86)	10.09 (9.94–10.24)
Wasting–Underweight	2.61 (2.49–2.73)	1.62 (1.54–1.71)	2.05 (1.97–2.12)
Stunting–Wasting–Underweight	2.61 (2.49–2.73)	1.46 (1.38–1.54)	1.95 (1.88–2.02)

Notes: CIAF = Composite Index of Anthropometric Failure.

**Table 3:** Random effect model and model fitness for the assessment of undernutrition among children in sub-Saharan Africa.

Random Variables		Null Model			Model I			Model II			Model III		
		Variance	PCV	ICC	Variance	PCV	ICC	Variance	PCV	ICC	Variance	PCV	ICC
Country	0–23	0.11	Ref	3.26	0.09	18	2.7	0.10	9	2.9	0.07	36	2.1
	24–59	0.25	Ref	7.1	0.22	12	6.3	0.21	16	6	0.19	24	5.5
Community	0–23	0.47	Ref	12.5	0.44	6	11.8	0.43	8	11.6	0.43	9	11.5
	24–59	0.59	Ref	15.2	0.43	27	11.6	0.45	24	12	0.43	27	11.5
Model Comparison													
LL	0–23	−71,889.364			−63,892.908			−71,274.554			−63,795.765		
	24–59	−95,254.045			−92,257.16			−94,146.259			−91,961.195		
Deviance	0–23	143,778.728			127,785.816			142,545.108			127,591.520		
	24–59	190,508.09			184,514.32			188,292.518			183,922.390		

Notes: ICC = intraclass correlation coefficient (%), LL = log likelihood, PCV = percentage change in variance (%) (change in variance after incorporating individual- and community-level factors with reference to variance of the null model). Model I contains individual-level variables, model II contains community-level variables, and model III contains both individual- and community-level variables.

**Table 4:** Determinants of standalone and coexisting forms of undernutrition in children aged 0–23 months in sub-Saharan Africa.

Independent Variables		Outcome Variable Categories					
		Stunting Only	Wasting Only	Underweight Only	SU	WU	SWU
		(aRRR with 95% CI)					
Sex of child	Male						
	Female	0.62 (0.58, 0.66) **	0.76 (0.69, 0.83) **	0.71 (0.59, 0.84) **	0.56 (0.51, 0.62) **	0.67 (0.59, 0.76) **	0.39 (0.34, 0.45) **
Age of the child in months	0–5						
	6–8	1.09 (0.91, 1.29)	0.88 (0.67, 1.16)	1.36 (0.93, 1.99)	1.04 (0.84, 1.28)	1.58 (1.16, 2.16) **	2.27 (1.43, 3.59) **
	9–23	2.8 (2.41, 3.25) **	0.74 (0.64, 0.86) **	1.11 (0.80, 1.55)	2.27 (1.86, 2.75) **	1.61 (1.19, 2.18) **	5.75 (3.82, 8.75) **
Birth weight	Low birth weight	1.49 (1.26, 1.77) **	1.13 (0.81, 1.57)	1.47 (1.03, 2.12) *	2.22 (1.77, 2.77) **	1.95 (1.42, 2.69) **	2.2 (1.52, 3.22) **
	Normal						
	Macrosomia	0.88 (0.77, 1.01)	0.83 (0.58, 1.17)	0.71 (0.50, 1.00)	0.77 (0.54, 1.08)	0.95 (0.69, 1.29)	0.74 (0.50, 1.12)
Type of birth	Single						
	Twin/multiple	2.56 (1.92, 3.42) **	1.23 (0.5, 3.00)	2.92 (1.76, 4.9) **	5.21 (4.09, 6.62) **	1.39 (0.76, 2.56)	7.32 (5.53, 9.68) **
Age-appropriate feeding	No						
	Yes	1.02 (0.9, 1.15)	1.08 (0.88, 1.32)	0.85 (0.64, 1.16)	0.79 (0.67, 0.93) **	0.83 (0.62, 1.09)	0.68 (0.48, 0.96) *
Birth order	First						
	2nd–4th	0.9 (0.82, 1.00)	0.99 (0.83, 1.19)	0.9 (0.75, 1.08)	0.81 (0.73, 0.91) **	0.94 (0.78, 1.14)	0.86 (0.69, 1.05)
	5th and above	0.95 (0.82, 1.1)	1.19 (0.89, 1.59)	0.85 (0.61, 1.18)	0.89 (0.77, 1.03)	0.95 (0.77, 1.17)	0.85 (0.66, 1.08)
Place of delivery	Home						
	Health facility	0.95 (0.87, 1.04)	0.86 (0.64, 1.16)	0.79 (0.63, 0.98) *	0.71 (0.63, 0.8) **	0.84 (0.72, 0.98) *	0.61 (0.5, 0.75) **
ANC	No						
	1–3	1.01 (0.87,1.16)	0.79 (0.62, 1.02)	0.93 (0.74, 1.19)	0.99 (0.81, 1.21)	0.8 (0.63, 1.00)	0.83 (0.63, 1.07)
	4 and above	0.86 (0.77, 0.97) *	0.85 (0.7, 1.04)	0.89 (0.66, 1.18)	0.84 (0.7, 1.00)	0.72 (0.57, 0.91) **	0.69 (0.53, 0.89) **
Post natal checkup	No						
	Yes	1.02 (0.92, 1.12)	1.02 (0.87, 1.19)	1.14 (0.93, 1.42)	0.85 (0.79, 0.92) **	1.15 (0.98, 1.36)	0.94 (0.8, 1.09)
Household wealth status	Poorest						
	Poorer	0.97 (0.9, 1.03)	0.87 (0.75, 1.04)	0.99 (0.79, 1.24)	0.86 (0.78, 0.95) **	0.9 (0.72, 1.13)	0.79 (0.69, 0.9) **
	Middle	0.78 (0.7, 0.88) **	0.89 (0.69, 1.09)	0.8 (0.63, 1.03)	0.71 (0.61, 0.84) **	0.91 (0.76, 1.09)	0.63 (0.49, 0.8) **
	Richer	0.74 (0.64, 0.85) **	0.91 (0.73, 1.15)	0.79 (0.58, 1.07)	0.57 (0.46, 0.71) **	0.77 (0.57, 1.04)	0.6 (0.44, 0.81) **
	Richest	0.46 (0.39, 0.55) **	0.92 (0.67, 1.24)	0.69 (0.42, 1.16)	0.36 (0.28, 0.44) **	0.66 (0.47, 0.93) *	0.4 (0.26, 0.62) **
Maternal educational level	No	1.11 (0.98, 1.25)	1.35 (1.02, 1.68) **	1.29 (0.92, 1.82)	1.48 (1.28, 1.69) **	1.46 (1.21, 1.73) **	1.73 (1.36, 2.2) **
	Primary	1.22 (1.11, 1.35) **	1.12 (0.9, 1.37)	1.25 (1.01, 1.52)*	1.33 (1.22, 1.48) **	1.05 (0.87, 1.27)	1.26 (0.98, 1.6)
	Secondary and above						
Maternal age	15–24						
	25–34	0.88 (0.82, 0.96) **	0.98 (0.81, 1.17)	1.07 (0.85, 1.36)	1.01 (0.92, 1.12)	1.1 (0.96, 1.27)	1.01 (0.84, 1.21)
	35–49	0.92 (0.8, 1.06)	1.06 (0.83, 1.37)	1.04 (0.76, 1.43)	1.02 (0.86, 1.19)	1.16 (0.92, 1.49)	1.06 (0.86, 1.32)
Maternal working status	Not working						
	Working	1.04 (0.96, 1.15)	0.85 (0.73, 0.99) *	0.89 (0.74, 1.08)	1.09 (0.98, 1.22)	0.87 (0.76, 1.01)	0.99 (0.86, 1.13)
Media exposure	No						
	Yes	0.94 (0.86, 1.02)	0.9 (0.75, 1.08)	1.15 (1.00, 1.34)	0.93 (0.84, 1.03)	1.10 (0.98, 1.26)	0.98 (0.86, 1.17)
Residence	Urban						
	Rural	1.05 (0.96, 1.15)	0.98 (0.78, 1.22)	0.95 (0.76, 1.19)	1.01 (0.91, 1.12)	0.85 (0.68, 1.05)	0.84 (0.68, 1.02)
Community illiteracy level	Low						
	High	0.88 (0.77, 1.02)	1.43 (1.14, 1.82) **	1.67 (1.32, 2.18) **	1.12 (0.97, 1.29)	1.47 (1.17, 1.87) **	1.35 (1.09, 1.68) **
Community poverty level	Low	0.98 (0.87, 1.09)	1.13 (0.86, 1.46)	0.96 (0.77, 1.19)	0.99 (0.92, 1.08)	0.97 (0.83, 1.14)	0.91 (0.75, 1.1)
	High						
Type of toilet facility	Unimproved						
	Improved	1.03 (0.94, 1.14)	0.92 (0.78, 1.07)	0.87 (0.69, 1.08)	0.98 (0.89, 1.07)	0.95 (0.83, 1.08)	0.98 (0.85, 1.15)
Year of survey	Before COVID-19						
	During COVID-19	0.77 (0.58, 1.02)	0.85 (0.59, 1.22)	0.77 (0.58, 1.02)	0.68 (0.50, 0.93) *	0.93 (0.69, 1.24)	0.66 (0.45, 0.94) *
Random component							
Variance			Intercorrelation coefficient (%)			Percentage change in variance (%)	
Country		Community	Country	Community		Country	Community
0.07		0.43	2.1	11.5		36	9

Notes: ANC = antenatal care, aRRR = adjusted relative risk ratio, SU = stunting–underweight, WU = wasting–underweight, SWU = stunting–wasting–underweight. * *p*-value < 0.05, ** *p*-value < 0.01.

**Table 5:** Determinants of standalone and coexisting forms of undernutrition in children aged 24–59 months in sub-Saharan Africa.

Independent Variables		Outcome Variable Categories					
		Stunting Only	Wasting Only	Underweight Only	SU	WU	SWU
		(aRRR with 95% CI)					
Sex of child	Male						
	Female	0.81 (0.77, 0.85) **	1.01 (0.89, 1.16)	1.36 (1.18, 1.58) **	0.89 (0.83, 0.96) **	0.85 (0.77, 0.93) **	0.65 (0.57, 0.74) **
Age of the child in months	24–35						
	36–47	0.82 (0.77, 0.87) **	0.74 (0.59, 0.91) **	0.95 (0.79, 1.14)	0.88 (0.82, 0.95) **	0.79 (0.61, 1.01)	0.64 (0.49, 0.83) **
	48–59	0.55 (0.51, 0.59) **	0.99 (0.79, 1.25)	1.03 (0.83, 1.28)	0.72 (0.62, 0.83) **	0.95 (0.75, 1.2)	0.45 (0.35, 0.58) **
Type of birth	Single birth						
	Multiple births	1.89 (1.67, 2.14) **	0.87 (0.50, 1.49)	1.24 (0.76, 2.03)	2.45 (1.91, 3.13) **	1.65 (1.13, 2.43) *	2.48 (1.71, 3.62) **
Birth order	First						
	2nd–4th	1.28 (0.78, 2.09)	0.73 (0.12, 4.55)	0.81 (0.21, 3.13)	0.93 (0.57, 1.51)	1.76 (0.78, 3.94)	1.45 (0.26, 8.23)
	5th and above	1.42 (0.86, 2.36)	0.60 (0.09, 4.13)	0.72 (0.19, 2.73)	1.02 (0.63, 1.65)	1.65 (0.67, 4.09)	1.52 (0.27, 8.69)
Birth interval	No prior birth						
	Less than 33	0.98 (0.61, 1.58)	1.34 (0.23, 7.93)	1.35 (0.33, 5.39)	1.57 (0.96, 2.56)	0.54 (0.23, 1.25)	1.15 (0.22, 5.96)
	33 and above	0.77 (0.47, 1.26)	1.06 (0.18, 6.35)	1.12 (0.28, 4.50)	1.06 (0.65, 1.72)	0.53 (0.22, 1.29)	0.77 (0.15, 3.93)
Maternal age	15–24						
	25–34	0.78 (0.71, 0.86) **	1.11 (0.85, 1.43)	1.08 (0.82, 1.42)	0.77 (0.7, 0.85) **	0.89 (0.77, 1.08)	0.83 (0.71, 0.99) *
	35–49	0.67 (0.56, 0.78) **	1.22 (0.82, 1.81)	1.12 (0.78, 1.66)	0.68 (0.58, 0.79) **	0.83 (0.63, 1.09)	0.76 (0.57, 1.00)
Maternal working status	Not working						
	Working	1.04 (0.95, 1.15)	0.76 (0.61, 0.93) *	0.79 (0.65, 0.96) *	0.96 (0.87, 1.06)	0.79 (0.66, 0.94) **	0.87 (0.75, 1.00)
Maternal educational level	No education	1.39 (1.21, 1.61) **	1.16 (0.82, 1.65)	1.07 (0.74, 1.55)	1.66 (1.42, 2.95) **	1.42 (1.19, 1.69) **	1.5 (1.21, 1.86) **
	Primary education	1.46 (1.31, 1.64) **	0.79 (0.55, 1.11)	0.98 (0.78, 1.23)	1.46 (1.26, 1.69) **	0.98 (0.82, 1.19)	1.08 (0.93, 1.30)
	Secondary and above						
Marital status	Not married						
	Married	0.84 (0.78, 0.9) **	1.08 (0.90, 1.30)	0.99 (0.77, 1.25)	0.84 (0.78, 0.92) **	0.90 (0.65, 1.25)	0.85 (0.65, 1.11)
Household wealth status	Poorest						
	Poorer	0.89 (0.82, 0.96) **	0.61 (0.45, 0.82) **	0.68 (0.56, 0.84) **	0.82 (0.76, 0.88) **	0.78 (0.60, 1.01)	0.72 (0.55, 0.94) *
	Middle	0.76 (0.69, 0.85) **	0.64 (0.51, 0.81) **	0.57 (0.44, 0.74) **	0.65 (0.59, 0.72) **	0.59 (0.48, 0.72) **	0.53 (0.37, 0.78) **
	Richer	0.59 (0.54, 0.64) **	0.56 (0.44, 0.72) **	0.50 (0.35, 0.74) **	0.47 (0.42, 0.53) **	0.55 (0.41, 0.73) **	0.42 (0.30, 0.58) **
	Richest	0.35 (0.31, 0.41) **	0.52 (0.39, 0.68) **	0.41 (0.25, 0.67) **	0.31 (0.26, 0.38) **	0.48 (0.29, 0.77) **	0.28 (0.17, 0.45) **
Media exposure	No						
	Yes	0.94 (0.88, 1.01)	1.1 (0.86, 1.42)	1.36 (1.01,1.79)	0.84 (0.78, 0.90) **	1.07 (0.84, 1.37)	0.78 (0.64, 0.95) *
Type of toilet facility	Unimproved						
	Improved	0.95 (0.88, 1.02)	1.02 (0.80, 1.29)	0.96 (0.79, 1.16)	0.91 (0.83, 0.99)*	1.00 (0.8, 1,24)	0.88 (0.74, 1.04)
Water source	Improved						
	Not improved	1.01 (0.92, 1.01)	0.98 (0.77, 1.24)	0.88 (0.67, 1.14)	0.93 (0.83, 1.05)	0.88 (0.68, 1.14)	0.89 (0.76, 1.05)
Community illiteracy level	Low						
	High	1.05 (0.89, 1.23)	1.81 (1.28, 2.55) **	1.82 (1.29, 2.57) **	1.34 (1.08, 1.65) **	1.74 (1.38, 2.19) **	1.73 (1.32, 2.27) **
Community poverty level	Low	0.9 (0.83, 0.98) *	1.08 (0.94, 1.24)	0.98 (0.83, 1.16)	1.00 (0.88, 1.14)	1.09 (0.9, 1.32)	0.95 (0.81, 1.12)
	High						
Place of residence	Urban						
	Rural	1.03 (0.93, 1.15)	0.78 (0.58, 1.05)	0.94 (0.72, 1.24)	1.12 (0.98, 1.27)	1.01 (0.79, 1.29)	0.92 (0.73, 1.17)
Year of survey	Before COVID-19						
	During COVID-19	0.63 (0.43, 0.93) *	1.24 (0.88, 1.74)	1.19 (0.83, 1.71)	0.64 (0.41, 0.98) *	1.29 (0.88, 1.90)	0.87 (0.56, 1.35)
Random component							
Variance			Intercorrelation coefficient (%)			Percentage change in variance (%)	
Country		Community	Country	Community		Country	Community
0.19		0.43	5.5	11.5		24	27

Notes: aRRR = adjusted relative risk ratio, SU = stunting–underweight, WU = wasting–underweight, SWU = stunting–wasting–underweight. * *p*-value < 0.05, ** *p*-value < 0.01.

## Data Availability

The authors do not have permission to share the data. However, all of the data used will be made available from the measure DHS program (https://www.dhsprogram.com/Methodology/Survey-Types/DHS.cfm (accessed on 25 November 2023)) upon reasonable request.

## Supplementary Materials

The following supporting information can be downloaded at: https://www.mdpi.com/article/10.3390/nu17020252/s1, Table S1: Model I contain individual level determinants of various standalone and coexisting undernutrition of children aged 0–23 months in sub-Saharan Africa; Table S2: Model II contain community level determinants of standalone and coexisting forms of undernutrition of children aged 0–23 month in sub-Saharan Africa; Table S3: Model I contain individual level determinants of standalone and coexisting forms of undernutrition of children aged 24–59 month in sub-Saharan Africa; Table S4: Model II contain community level determinants of standalone and coexisting forms of undernutrition of children aged 24–59 month in sub-Saharan Africa.

## References

[1] United Nations International Children’s Emergency Fund (UNICEF)/World Health Organization (WHO)/World Bank (WB) (2023). Levels and Trends in Child Malnutrition Child Malnutrition: UNICEF/WHO/World Bank Group Joint Child Malnutrition Estimates.

[2] Katti A., Talawar K., Kadlimatti M., Kumar V. (2021). The co-morbidities associated with protein energy malnutrition in children. Eur. J. Mol. Clin. Med..

[3] Black R.E., Talawar K., Kadlimatti M., Kumar V. (2013). Maternal and child undernutrition and overweight in low-income and middle-income countries. Lancet.

[4] Khan S., Zaheer S., Safdar N.F. (2019). Determinants of stunting, underweight and wasting among children< 5 years of age: Evidence from 2012–2013 Pakistan demographic and health survey. BMC Public Health.

[5] Nandy S., Svedberg P. (2012). The Composite Index of Anthropometric Failure (CIAF): An alternative indicator for malnutrition in young children. Handbook of Anthropometry: Physical Measures of Human Form in Health and Disease.

[6] Chikhungu L.C. (2022). Trends and patterns of stunted only and stunted underweight children in Malawi: A confirmation for child nutrition practitioners to continue focusing on stunting. Malawi Med. J..

[7] Bhattacharyya A.K. (2006). Composite index of anthropometric failure (CIAF) classification: Is it more useful?. Bull. World Health Organ..

[8] Anato A. (2022). Predictors of wasting among children under-five years in largely food insecure area of north Wollo, Ethiopia: A cross-sectional study. J. Nutr. Sci..

[9] Sunguya B.F., Zhu S., Mpembeni R., Huang J. (2019). Trends in prevalence and determinants of stunting in Tanzania: An analysis of Tanzania demographic health surveys (1991–2016). Nutr. J..

[10] Sen J., Mondal N. (2012). Socio-economic and demographic factors affecting the Composite Index of Anthropometric Failure (CIAF). Ann. Hum. Biol..

[11] Chowdhury M.R.K., Khan H.T., Rashid M., Kabir R., Islam S., Islam M.S., Kader M. (2021). Differences in risk factors associated with single and multiple concurrent forms of undernutrition (stunting, wasting or underweight) among children under 5 in Bangladesh: A nationally representative cross-sectional study. BMJ Open.

[12] Choudhury N., Raihan M.J., Sultana S., Mahmud Z., Farzana F.D., Haque M.A., Rahman A.S., Waid J.L., Chowdhury A.M.R., Black R.E. (2017). Determinants of age-specific undernutrition in children aged less than 2 years—The Bangladesh context. Matern. Child Nutr..

[13] Adekanmbi V.T., Kayode G.A., Uthman O.A. (2013). Individual and contextual factors associated with childhood stunting in Nigeria: A multilevel analysis. Matern. Child Nutr..

[14] Rutstein S.O., Rojas G. (2006). Guide to DHS Statistics.

[15] UNICEF (2022). Conceptual Frameworkon Maternal and Child Nutrition.

[16] Seifu B.L., Mare K.U., Legesse B.T., Tebeje T.M. (2024). Double burden of malnutrition and associated factors among women of reproductive age in sub-Saharan Africa: A multilevel multinomial logistic regression analysis. BMJ Open.

[17] Islam M.S., Biswas T. (2020). Prevalence and correlates of the composite index of anthropometric failure among children under 5 years old in Bangladesh. Matern. Child Nutr..

[18] Otorkpa O.J., Yusuf A.M., Aborode A.T. (2024). Climate and conflict-induced child nutrition crisis in Sub-Saharan Africa. Confl. Health.

[19] Arhin J. (2019). Socio-Cultural Factors Influencing Malnutrition Among Children Under-Five Years in the Cape Coast Metropolis.

[20] Amadu I., Seidu A.A., Duku E., Frimpong J.B., Jnr J.E.H., Aboagye R.G., Adu C., Ahinkorah B.O. (2021). Risk factors associated with the coexistence of stunting, underweight, and wasting in children under 5 from 31 sub-Saharan African countries. BMJ Open.

[21] Khaliq A., Wraith D., Miller Y., Nambiar-Mann S. (2021). Prevalence, trends, and socioeconomic determinants of coexisting forms of malnutrition amongst children under five years of age in Pakistan. Nutrients.

[22] Bhusal U.P., Sapkota V.P. (2022). Socioeconomic and demographic correlates of child nutritional status in Nepal: An investigation of heterogeneous effects using quantile regression. Glob. Health.

[23] Khanam M., Shimul S.N., Sarker A.R. (2019). Individual-, household-, and community-level determinants of childhood undernutrition in Bangladesh. Health Serv. Res. Manag. Epidemiol..

[24] Ziba M., Kalimbira A.A. (2018). Predictors of Anthropometric Failure Among Malawian Children Less Than 5 Years of Age. J. Nutr. Ecol. Food Res..

[25] Adedokun S.T., Yaya S. (2021). Factors associated with adverse nutritional status of children in sub-Saharan Africa: Evidence from the Demographic and Health Surveys from 31 countries. Matern. Child Nutr..

[26] Atukunda P., Eide W.B., Kardel K.R., Iversen P.O., Westerberg A.C. (2021). Unlocking the potential for achievement of the UN Sustainable Development Goal 2–‘Zero Hunger’–in Africa: Targets, strategies, synergies and challenges. Food Nutr. Res..

[27] Engebretsen I.M.S., Tylleskär T., Wamani H., Karamagi C., Tumwine J.K. (2008). Determinants of infant growth in Eastern Uganda: A community-based cross-sectional study. BMC Public Health.

[28] Thurstans S., Opondo C., Seal A., Wells J., Khara T., Dolan C., Briend A., Myatt M., Garenne M., Sear R. (2020). Boys are more likely to be undernourished than girls: A systematic review and meta-analysis of sex differences in undernutrition. BMJ Glob. Health.

[29] Pillai V.K., Ortiz-Rodriguez J. (2015). Child malnutrition and gender preference in India: The role of culture. Health Sci. J..

[30] Fentahun N., Belachew T., Lachat C. (2016). Determinants and morbidities of multiple anthropometric deficits in southwest rural Ethiopia. Nutrition.

[31] Kandala N.-B., Emina J.B., Nzita P.D.K., Cappuccio F.P. (2009). Diarrhoea, acute respiratory infection, and fever among children in the Democratic Republic of Congo. Soc. Sci. Med..

[32] Fenske N., Burns J., Hothorn T., Rehfuess E.A. (2013). Understanding Child Stunting in India: A Comprehensive Analysis of Socio-Economic, Nutritional and Environmental Determinants Using Additive Quantile Regression. PLoS ONE.

[33] Kebede D., Aynalem A. (2021). Prevalence of undernutrition and potential risk factors among children below five years of age in Somali region, Ethiopia: Evidence from 2016 Ethiopian demographic and health survey. BMC Nutr..

[34] Ntenda P.A.M. (2019). Association of low birth weight with undernutrition in preschool-aged children in Malawi. Nutr. J..

[35] Goyena E.A., Maniego M.L.V. (2022). Adherence to age-appropriate feeding practices among Filipino children under two: An analysis of the 2018- 2019 Expanded National Nutrition Survey. Malays. J. Nutr..

[36] WHO (2009). Infant and Young Child Feeding: Model Chapter for Textbooks for Medical Students and Allied Health Professionals..

[37] Keats A. (2018). Women's schooling, fertility, and child health outcomes: Evidence from Uganda's free primary education program. J. Dev. Econ..

[38] Fagbamigbe A.F., Uthman A.O., Ibisomi L. (2021). Hierarchical disentanglement of contextual from compositional risk factors of diarrhoea among under-five children in low- and middle-income countries. Sci. Rep..

