# Supplementary material for: Risk Factors of Standalone and Coexisting Forms of Undernutrition Among Children in Sub-Saharan Africa: A Study Using Data from 26 Country-Based Demographic and Health Surveys
Source: Nutrients. 2025 Jan 11;17(2):252. doi: 10.3390/nu17020252 (PMC11767797; doi:10.3390/nu17020252)
Supplement: Supplementary file 1 [file nutrients-17-00252-s001.zip › Suplementary file 1.docx]

Supplementary files containing multilevel multinomial logistic regression analyses results of model I(with individual level factors) and model II (with community level factors) for children aged 0-23 months and 24-59 months in sub-Saharan Africa.

**Table S1. Model I contain individual level determinants of various standalone and coexisting undernutrition of children aged 0-23 months in sub-Saharan Africa.**

| Independent variables | | | Outcome variable categories | | | | | | | | | |
| --- | --- | --- | --- | --- | --- | --- | --- | --- | --- | --- | --- | --- |
|  |  |  | **Stunting only** | | **Wasting only** | **Underweight only** | | **SU** | | **WU** | | **SWU** |
|  |  |  | **(aRRR with 95%CI)** | | | | | | | | | |
| Sex of child | Male | |  | |  |  | |  | |  | |  |
|  | Female | | 0.62(0.58, 0.67) | | 0.76(0.69, 0.83) | 0.71(0.59, 0.84) | | 0.59(0.51, 0.63) | | 0.66(0.59, 0.76) | | 0.39(0.34, 0.45) |
| Age | 0-5 | |  | |  |  | |  | |  | |  |
|  | 6-8 | | 1.08(0.91, 1.29) | | 0.87(0.66, 1.16) | 1.34(0.91, 1.99) | | 1.04(0.84, 1.29) | | 1.55(1.17, 2.16)** | | 2.29(1.43, 3.63)** |
|  | 9-23 | | 2.8(2.41, 3.25)** | | 0.74(0.64, 0.87)** | 1.08(0.77, 1.54 | | 2.27(1.85, 2.77)** | | 1.58(1.16, 2.16)** | | 5.75(3.78, 8.75)** |
| Birth weight | Low birth weight | | 1.47(1.24, 1.75)** | | 1.13(0.81, 1.57) | 1.47(1.03, 2.12)* | | 2.2(1.77, 2.74)** | | 1.97(1.42, 2.72)** | | 2.2(1.52, 3.18)** |
|  | Normal | |  | |  |  | |  | |  | |  |
|  | Macrosomic | | 0.88(0.77, 1.01) | | 0.83(0.58, 1.17) | 0.71(0.5, 0.99)* | | 0.77(0.54, 1.08) | | 0.94(0.69, 1.28) | | 0.76(0.51, 1.12) |
| Type of birth | Single | |  | |  |  | |  | |  | |  |
|  | Twin/multiple | | 2.55(1.91, 3.42)** | | 1.23(0.51, 3.00) | 2.92(1.73, 4.85)** | | 5.15(4.09, 6.55)** | | 1.40(0.76, 2.55) | | 7.24(5.47, 9.58)** |
| Age-appropriate feeding | No | |  | |  |  | |  | |  | |  |
|  | Yes | | 1.02(0.9, 1.16) | | 1.07(0.87, 1.32) | 0.86(0.63, 1.16) | | 0.79(0.68, 0.95)* | | 0.8(0.60, 1.08) | | 0.69(0.49, 0.97)* |
| Birth order | First | |  | |  |  | |  | |  | |  |
|  | 2^nd^-4^th^ | | 0.9(0.82, 1.00) | | 0.99(0.83, 1.18) | 0.89(0.74, 1.08) | | 0.81(0.73, 0.91)* | | 0.94(0.78, 1.14) | | 0.86(0.69, 1.06) |
|  | 5^th^ and above | | 0.95(0.81, 1.12) | | 1.18(0.89, 1.57) | 0.84(0.61, 1.17) | | 0.89(0.78, 1,03) | | 0.94(1.34, 1.17) | | 0.86(0.66, 1.12) |
| Place of delivery | Home | |  | |  |  | |  | |  | |  |
|  | Health facility | | 0.95(0.86, 1.04) | | 0.86(0.64, 1.16) | 0.77(0.63, 0.96)* | | 0.7(0.62, 0.81)** | | 1.19(0.71, 0.99)* | | 0.60(0.49, 0.75)** |
| ANC | No | |  | |  |  | |  | |  | |  |
|  | 1-3 | | 1.02(0.88, 1.17) | | 0.79(0.62, 1.10) | 0.93(0.75, 1.16) | | 0.98(0.79, 1.19) | | 0.81(0.64, 1.02) | | 0.80(0.61, 1.04) |
|  | 4 and above | | 0.87(0.77, 0.98)* | | 0.85(0.7, 1.02) | 0.86(0.65, 1.14) | | 0.83(0.69, 0.98)* | | 0.73(0.56, 0.92)** | | 0.68(0.52, 0.87)** |
| Post natal checkup | No | |  | |  |  | |  | |  | |  |
|  | Yes | | 1.02(0.92, 1,13) | | 1.02(0.86, 1.22) | 1.14(0.91, 1.4) | | 0.85(0.79, 0.92)** | | 1.16(0.97, 1.4) | | 0.94(0.80, 1.08) |
| Wealth status | Poorest | |  | |  |  | |  | |  | |  |
|  | Poorer | | 0.96(1.17, 1.02) | | 0.88(0.76, 1.05) | 0.99(0.80, 1.23) | | 0.86(0.7, 0.96) | | 0.89(0.72, 1.12) | | 0.79(0.69, 0.89)** |
|  | Middle | | 0.77(0.69, 0.85)** | | 0.93(0.76, 0.87) | 0.79(0.63, 0.99)* | | 0.72(0.62, 0.84)** | | 0.89(0.74, 1.06) | | 0.62(0.51, 0.77)** |
|  | Richer | | 0.71(0.62, 0.82)** | | 0.98(0.75, 1.27) | 0.77(0.61, 0.99)* | | 0.57(0.47, 0.70)** | | 0.77(0.57, 1.04) | | 0.61(0.47, 0.78)** |
|  | Richest | | 0.44(0.36, 0.53)** | | 0.98(0.75, 1.27) | 0.68(0.47, 0.98)* | | 0.36(0.29, 0.44)** | | 0.67(0.48, 0.93)* | | 0.42(0.29, 0.60)** |
| Maternal education | No | | 1.01(0.85, 1.19) | | 1.63(1.31, 2.03)** | 1.75(1.27, 2.41)** | | 1.58(1.34, 1.85)** | | 1.75(1.42, 2.14)** | | 2.03(1.58, 2.61)** |
|  | Primary | | 1.22(1.09, 1.36)** | | 1.14(0.89, 1.46) | 1.29(1.05, 1.59)* | | 1.36(1.23, 1.51)** | | 1.06(0.88, 1.28) | | 1.27(0.98, 1.63) |
|  | Secondary and above | |  | |  |  | |  | |  | |  |
| Maternal age | 15-24 | |  | |  |  | |  | |  | |  |
|  | 25-34 | | 0.88(0.82, 0.97)** | | 0.98(0.81, 1.16) | 1.06(0.83, 1.36) | | 1.34(0.91, 1.11) | | 1.11(0.95, 1.28) | | 0.99(0.83, 1.18) |
|  | 35-49 | | 0.94(0.79, 1.09) | | 1.05(0.83, 1.33) | 1.01(0.72, 1.43) | | 1.00(0.85, 1.17) | | 1.17(0.91, 1.5) | | 1.03(0.83, 1.28) |
| Maternal working status | Not working | |  | |  |  | |  | |  | |  |
|  | Working | | 1.04(0.96, 1.14) | | 0.83(0.71, 0.99)* | 0.9(0.73, 1.11) | | 1.11(0.99, 1.24) | | 0.85(0.73, 0.99)* | | 1.00(0.85, 1.17) |
| Media exposure | No | |  | |  |  | |  | |  | |  |
|  | Yes | | 0.94(0.86, 1.02) | | 0.9(0.73, 1.11) | 1.14(0.99, 1.32) | | 0.92(0.83, 1.02) | | 1.12(0.98, 1.28) | | 0.97(0.84, 1.1) |
| Type of toilet facility | Unimproved | |  | |  |  | |  | |  | |  |
|  | Improved | | 1.04(0.94, 1.15) | | 0.92(0.78, 1.07) | 0.85(0.69, 1.05) | | 0.97(0.88, 1.06) | | 0.96(0.84, 1.08) | | 0.98(0.84, 1,14) |
| Random component | | | | | | | | | | | | |
| Variance | | | | **Intercorrelation coefficient** | | | | | **Percentage change in variance** | | | |
| Country | | Cluster | | Country | | | Cluster | | Country | | Cluster | |
| 0.09 | | 0.44 | | 2.7 | | | 11.8 | | 18 | | 6 | |

**SU**=Stunting-Underweight,  **WU**=Wasting-Underweight, **SWU**=Stunting-Wasting-Underweight, **aRRR**= adjusted relative risk ratio

*p-vale<0.05

**p-value<0.01

**Table S2. Model II contain community level determinants of standalone and coexisting forms of undernutrition of children aged 0-23 month in sub-Saharan Africa**

| Independent variables | | | Outcome variable categories | | | | | | | | | |
| --- | --- | --- | --- | --- | --- | --- | --- | --- | --- | --- | --- | --- |
|  |  |  | **Stunting only** | | **Wasting only** | | **Underweight only** | | **SU** | **WU** | | **SWU** |
|  |  |  | **(aRRR with 95%CI)** | | | | | | | | | |
| Community illiteracy level | Low | |  | |  |  | | |  |  | |  |
|  | High | | 0.92(0.77, 1.09) | | 1.78(1.39, 2.29)** | 1.95(1.52, 2.51)** | | | 1.42(1.16, 1.75)** | 1.92(1.48, 2.48)** | | 1.95(1.46, 2.61)** |
| Community poverty level | Low | | 0.76(0.69, 0.84)** | | 0.96(0.80, 1.15) | 0.78(0.68, 0.89)** | | | 0.67(0.62, 0.73)** | 2.23(0.67, 0.95)* | | 0.63(0.52, 0.76)** |
|  | High | |  | |  |  | | |  |  | |  |
| Place of residence | Urban | |  | |  |  | | |  |  | |  |
|  | Rural | | 1.32(1.19, 1.46)** | | 1.07(0.82, 1.40) | 1.08(0.91, 1.27) | | | 1.45(1.25, 1.69)** | 0.97(0.78, 1.20) | | 0.19(0.97, 1.45) |
| Survey year | Before covid | |  | |  |  | | |  |  | |  |
|  | During covid | | 0.82(0.60, 1.05) | | 0.85(0.58, 1.24) | 0.83(0.62, 1.10) | | | 0.70(0.52, 0.97)* | 0.94(0.70, 1.27) | | 0.68(0.47, 0.96)* |
| Random component | | | | | | | | | | | | |
| Variance | | | | **Intercorrelation coefficient(ICC)** | | | | | | **Percentage change in variance(PCV)** | | |
| Country | | Cluster | | Country | | | | Cluster | | Country | Cluster | |
| 0.1 | | 0.43 | | 2.9 | | | | 11.6 | | 9 | 8 | |

SU= Stunting-Underweight, WU= Wasting-Underweight, SWU=Stunting-Wasting-Underweight

*p-vale<0.05

**p-value<0.01

**Table S3. Model I contain individual level determinants of standalone and coexisting forms of undernutrition of children aged 24-59 month in sub-Saharan Africa**

| Independent variables | | | Outcome variable categories | | | | | | | | |
| --- | --- | --- | --- | --- | --- | --- | --- | --- | --- | --- | --- |
|  |  |  | **Stunting only** | | **Wasting only** | **Underweight only** | | **SU** | **WU** | | **SWU** |
|  |  |  | **(aRRR with 95%CI)** | | | | | | | | |
| Sex of child | Male | |  | |  |  | |  |  | |  |
|  | Female | | 0.81(0.78, 0.85)** | | 1.01(0.88, 1.15) | 1.36(1.17, 1.58)** | | 0.89(0.83, 0.97)** | 0.84(0.77, 0.93)** | | 0.65(0.57, 0.74)** |
| Child age | 24-35 | |  | |  |  | |  |  | |  |
|  | 36-47 | | 0.83(0.77, 0.87)** | | 0.74(0.59, 0.91)** | 0.95(0.79, 1.14) | | 0.88(0.82, 0.94)** | 0.78(0.61, 1.01) | | 0.64(0.49, 0.83)** |
|  | 48-59 | | 0.55(0.51, 0.59)** | | 0.98(0.79, 1.23) | 1.02(0.83, 1.27) | | 0.73(0.63, 0.84)** | 0.94(0.75, 1.18) | | 0.45(0.35, 0.57)** |
| Type of birth | Single birth | |  | |  |  | |  |  | |  |
|  | Multiple births | | 1.87(1.66, 2.12)** | | 0.9(0.52, 1.55) | 1.28(0.79, 2.07) | | 2.42(1.89, 3.09)** | 1.71(1.17, 2.47)** | | 2.52(1.73, 3.68)** |
| Birth order | 1^st^ birth order | |  | |  |  | |  |  | |  |
|  | 2^nd^ -4^th^ | | 1.29(0.79, 2.09) | | 0.68(0.11, 4.30) | 0.78(0.19, 3.04) | | 0.93(0.57, 1.52) | 1.68(0.73, 3.85) | | 1.43(0.26, 7.98) |
|  | 5^th^ and above | | 1.44(0.88, 2.38) | | 0.54(0.08, 3.78) | 0.66(0.17, 2.57) | | 1.03(0.64, 1.67) | 1.52(0.61, 3.78) | | 1.49(0.26, 8.39) |
| Birth interval | No prior birth | |  | |  |  | |  |  | |  |
|  | Less than 33 | | 0.98(0.61, 1.57 ) | | 1.41(0.24, 8.31) | 1.39(0.33, 5.73) | | 1.57(0.96, 2.56) | 0.55(0.23, 1.32) | | 1.16(0.23, 5.95) |
|  | 33 and above | | 0.77(0.47, 1.25) | | 1.12(0.18, 6.82) | 1.16(0.28, 4.74) | | 1.05(0.65, 1.71) | 0.56(0.22, 1.38) | | 0.78(0.16, 3.88) |
| Maternal age | 15-24 | |  | |  |  | |  |  | |  |
|  | 25-34 | | 0.78(0.71, 0.86)** | | 1.12(0.86, 1.46) | 1.08(0.81, 1.44) | | 0.77(0.67, 0.85)** | 0.9(0.73, 1.11) | | 0.83(0.7, 0.98)* |
|  | 35-49 | | 0.66(0.56, 0.78)** | | 1.27(0.86, 1.86) | 1.15(0.76, 1.73) | | 0.67(0.56, 0.79)** | 0.87(0.65, 1.15) | | 0.75(0.56, 0.99) |
| Working status | Not working | |  | |  |  | |  |  | |  |
|  | Working | | 1.05(0.96, 1.15) | | 0.71(0.57, 0.88)** | 0.74(0.59, 0.92)** | | 0.97(0.87, 1.07) | 0.74(0.59, 0.92)** | | 0.85(0.72, 1.01) |
| Educational level | No education | | 1.37(1.13, 1.67)** | | 1.43(0.98, 2.08) | 1.36(0.97, 1.89) | | 1.93(1.56, 2.37)** | 1.74(1.29, 2.35)** | | 1.90(1.43, 2.53)** |
|  | Primary education | | 1.46(1.29, 1.65)** | | 0.78(0.53, 1.14) | 0.99(0.79, 1.26) | | 1.49(1.29, 1.74)** | 1.00(0.79, 1.26) | | 1.10(0.91, 1.33) |
|  | Secondary and above | |  | |  |  | |  |  | |  |
| Marital status | Not married | |  | |  |  | |  |  | |  |
|  | Married | | 0.84(0.78, 0.91)** | | 1.04(0.86, 1.26) | 0.96(0.77, 1.23) | | 0.86(0.79, 0.93)** | 0.89(0.63, 1.25) | | 0.84(0.64, 1.11) |
| Wealth status | Poorest | |  | |  |  | |  |  | |  |
|  | Poorer | | 0.88(0.81, 0.95)** | | 0.59(0.42, 0.82)** | 0.66(0.53, 0.81)** | | 0.82(0.76, 0.88)** | 0.74(0.56, 0.98)* | | 0.7(0.53, 0.93)* |
|  | Middle | | 0.73(0.66, 0.81)** | | 0.64(0.48, 0.83)** | 0.53(0.42, 0.67)** | | 0.65(0.59, 0.72)** | 0.57(0.48, 0.68)** | | 0.51(0.35, 0.75)** |
|  | Richer | | 0.55(0.49, 0.62)** | | 0.57(0.43, 0.76)** | o.46(0.31, 0.68)** | | 0.47(0.41, 0.53)** | 0.52(0.36, 0.74)** | | 0.39(0.28, 0.57)** |
|  | Richest | | 0.33(0.28, 0.39)** | | 0.52(0.38, 0.72)** | 0.35(0.22, 0.56)** | | 0.3(0.25, 0.36)** | 0.42(0.26, 0.69)** | | 0.26(0.15, 0.42)** |
| Media exposure | No | |  | |  |  | |  |  | |  |
|  | Yes | | 0.93(0.87, 0.99)* | | 1.16(0.91, 1.47) | 1.39(1.03, 1.89)* | | 0.82(0.76, 0.88)** | 1.12(0.89, 1.40) | | 0.79(0.65, 0.96)* |
| Type of toilet facility | Unimproved | |  | |  |  | |  |  | |  |
|  | Improved | | 0.94(0.87, 1.02) | | 1.04(0.79, 1.36) | 0.96(0.78, 1.18) | | 0.88(0.81, 0.97)** | 1.01(0.83, 1.23) | | 0.87(0.73, 1.03) |
| Water source | improved | |  | |  |  | |  |  | |  |
|  | Not improved | | 1.03(0.94, 1,13) | | 0.92(0.69, 1.2) | 0.85(0.63, 1.13) | | 0.95(0.84, 1.08) | 0.85(0.63, 1.15) | | 0.89(0.75, 1.05) |
| Random component | | | | | | | | | | | |
| Variance | | | | **Intercorrelation coefficient(ICC)** | | | | | **Percentage change in variance(PCV)** | | |
| Country | | Cluster | | Country | | | Cluster | | Country | Cluster | |
| 0.23 | | 0.43 | | 6.3 | | | 11.6 | | 12 | 27 | |

SU= Stunting-Underweight, WU= Wasting-Underweight, SWU=Stunting-Wasting-Underweight

*p-vale<0.05

**p-value<0.01

**Table S4. Model II contain community level determinants of standalone and coexisting forms of undernutrition of children aged 24-59 month in sub-Saharan Africa**

| Independent variables | | | | Outcome variable categories | | | | | | | | | | | |
| --- | --- | --- | --- | --- | --- | --- | --- | --- | --- | --- | --- | --- | --- | --- | --- |
|  |  |  |  | **Stunting only** | | **Wasting only** | | **Underweight only** | | **SU** | | **WU** | | **SWU** | |
|  |  |  |  | **(aRRR with 95%CI)** | | | | | | | | | | | |
| Community illiteracy level | Low | |  | |  |  | | |  | |  | |  | |  |
|  | High | | 1.21(0.96, 1.51) | | 2.2(1.59, 3.03)** | 1.98(1.49, 2.65)** | | | 1.78(1.33, 2.38)** | | 2.22(1.71, 2.89)** | | 2.49(1.79, 3.46)** | |  |
| Community poverty level | Low | | 0.64(0.56, 0.71)** | | 0.9(0.78, 1.04) | 0.77(0.64, 0.92)** | | | 0.63(0.54, 0.75)** | | 0.84(0.68, 1.03) | | 0.56(0.46, 0.70)** | |  |
|  | High | |  | |  |  | | |  | |  | |  | |  |
| Place of residence | Urban | |  | |  |  | | |  | |  | |  | |  |
|  | Rural | | 1.47(1.31, 1.66)** | | 0.85(0.65, 1.12) | 1.07(0.83, 1.38) | | | 1.65(1.41, 1.95)** | | 1.15(0.91, 1.45) | | 1.32(1.12, 1.55)** | |  |
| Survey year | Before covid | |  | |  |  | | |  | |  | |  | |  |
|  | During covid | | 0.65(0.44, 0.9)* | | 1.32(0.95, 1.83) | 1.29(0.92, 1.80) | | | 0.65(0.43, 0.99) | | 1.37(0.94, 2.00) | | 0.91(0.59, 1.40) | |  |
| Random component | | | | | | | | | | | | | | | |
| Variance | | | | | | **Intercorrelation coefficient(ICC)** | | | | | | **Percentage change in variance(PCV)** | | | |
| Country | | | | Cluster | | Country | | | | Cluster | | Country | | Cluster | |
| 0.21 | | | | 0.45 | | 6 | | | | 12 | | 16 | | 24 | |
